# Meconium Microbiome of Very Preterm Infants across Germany

**DOI:** 10.1128/msphere.00808-21

**Published:** 2022-01-12

**Authors:** Jonas Klopp, Pamela Ferretti, Claudius U. Meyer, Katja Hilbert, Annette Haiß, Janina Marißen, Philipp Henneke, Hannes Hudalla, Sabine Pirr, Dorothee Viemann, Michael Zemlin, Sofia Kirke Forslund, Christoph Härtel, Peer Bork, Stephan Gehring, Thea Van Rossum, and the PRIMAL Consortium

**Affiliations:** a University Medical Center of the Johannes Gutenberg University Mainz, Mainz, Germany; b Structural and Computational Biology Unit, European Molecular Biology Laboratory (EMBL), Heidelberg, Germany; c Department of Pediatrics, University of Lübeck, Lübeck, Germany; d Department of Pediatrics, University of Würzburg, Würzburg, Germany; e Center for Pediatrics and Adolescent Medicine Pediatrics, Medical Center and Faculty of Medicine, University of Freiburg, Freiburg, Germany; f Institute for Immunodeficiency, Medical Center and Faculty of Medicine, University of Freiburg, Freiburg, Germany; g Center for Chronic Immunodeficiency (CCI) Medical Center and Faculty of Medicine, University of Freiburg, Freiburg, Germany; h Department of Neonatology, University of Heidelberg, Heidelberg, Germany; i Hanover Medical School, Department of Pediatric Pneumology, Allergology and Neonatology, Hanover, Germany; j Department of Pediatrics, Saar University Homburg, Homburg, Germany; k Experimental and Clinical Research Center, a cooperation of Charité-Universitätsmedizin and the Max-Delbrück Center, Berlin, Germany; l Max Delbrück Centre for Molecular Medicine, Berlin, Germany; m Charité – Universitätsmedizin Berlin, Berlin, Germany; n German Centre for Cardiovascular Research (DZHK), partner site Berlin, Berlin, Germany; o Yonsei Frontier Lab (YFL), Yonsei University, Seoul, South Korea; p Department of Bioinformatics, Biocenter, University of Würzburg, Würzburg, Germany; University of Michigan-Ann Arbor

**Keywords:** meconium, microbiome, 16S rRNA gene sequencing, mitochondria, mother

## Abstract

Meconium constitutes infants' first bowel movements postnatally. The consistency and microbial load of meconium are different from infant and adult stool. While recent evidence suggests that meconium is sterile *in utero*, rapid colonization occurs after birth. The meconium microbiome has been associated with negative health outcomes, but its composition is not well described, especially in preterm infants. Here, we characterized the meconium microbiomes from 330 very preterm infants (gestational ages 28 to 32 weeks) from 15 hospitals in Germany and in fecal samples from a subset of their mothers (N = 217). Microbiome profiles were compiled using 16S rRNA gene sequencing with negative and positive controls. The meconium microbiome was dominated by *Bifidobacterium*, Staphylococcus, and *Enterococcus* spp. and was associated with gestational age at birth and age at sample collection. Bifidobacterial abundance was negatively correlated with potentially pathogenic genera. The amount of bacterial DNA in meconium samples varied greatly across samples and was associated with the time since birth but not with gestational age or hospital site. In samples with low bacterial load, human mitochondrial sequences were highly amplified using commonly used, bacterial-targeted 16S rRNA primers. Only half of the meconium samples contained sufficient bacterial material to study the microbiome using a standard approach. To facilitate future meconium studies, we present a five-level scoring system (“MecBac”) that predicts the success of 16S rRNA bacterial sequencing for meconium samples. These findings provide a foundational characterization of an understudied portion of the human microbiome and will aid the design of future meconium microbiome studies.

**IMPORTANCE** Meconium is present in the intestines of infants before and after birth and constitutes their first bowel movements postnatally. The consistency, composition and microbial load of meconium is largely different from infant and adult stool. While recent evidence suggests that meconium is sterile *in utero*, rapid colonization occurs after birth. The meconium microbiome has been associated with short-term and long-term negative health outcomes, but its composition is not yet well described, especially in preterm infants. We provide a characterization of the microbiome structure and composition of infant meconium and maternal feces from a large study cohort and propose a method to evaluate meconium samples for bacterial sequencing suitability. These findings provide a foundational characterization of an understudied portion of the human microbiome and will aid the design of future meconium microbiome studies.

The development and composition of the early gut microbiome has been shown to impact the well-being of infants (1). This has been well-studied for infants of 2 to 46 months of life (2). However, the earliest developmental stages of the gut microbiome are still a topic of ongoing investigation (3, 4). Recent studies suggest that the uterus and thus the fetal meconium is sterile in healthy women (5). Sterility of fetal meconium was confirmed by sequencing and culturing stool samples obtained from the infant colon during elective breech cesarean deliveries (6). While it was previously thought that the first pass stool of infants after birth does not contain viable bacteria, recent studies, including the aforementioned, have found evidence of bacterial DNA in first pass meconium with the help of next-generation molecular techniques (6–9). First pass meconium is likely colonized with bacteria postnatally from breast milk, maternal gut and skin, and other environmental sources (10). Other studies have suggested that the uterus is not sterile (11) and that the post-birth meconium microbiome might mirror the *in utero* microbial environment (12, 13). Irrespective of whether meconium is colonized before, during or after birth, the composition and structure of early-life gut communities may impact health later in life (14).

Further explorations of associations between meconium microbiome composition and infant health require baseline characterization of the meconium microbiome. To date, few studies have investigated the meconium microbiome of newborns in sufficiently powered studies and with adequate controls. One noteworthy case-control trial from China investigated the association between neonatal jaundice and the meconium microbiome in 301 newborns (15) and found a higher abundance of Bifidobacterium pseudolongum and higher alpha diversity to be associated with a lower risk of jaundice in cesarean-born infants. They also discovered that meconium was mainly composed of bacteria from the phyla *Firmicutes*, *Proteobacteria*, *Actinobacteria*, *Cyanobacteria* and *Bacteroidetes*. The same primary taxa were identified in other meconium studies albeit in different relative proportions (16–18). However, many of these studies lack suitable controls. Studies are furthermore hampered by low bacterial DNA quantities in meconium compared to stool from older infants (1). Working with meconium thus requires the rigorous use of negative and positive controls to ensure reliable results (19, 20).

In this study, we provide a characterization of the microbiome in meconium samples of a representative cohort of preterm infants and their respective mothers from 15 hospitals in Germany as part of the “Priming Immunity at the beginning of life” (PRIMAL) study (21). We further elaborate on the problem of co-amplification of human mitochondrial 12S rRNA genes (the homologue of the 16S rRNA gene in prokaryotes [22]) that can arise while dealing with meconium samples and present a scoring system to aid the design of future meconium microbiome studies.

## RESULTS

### Sequencing and quantification of bacterial and mitochondrial amplicons.

A total of 547 samples were sequenced and analyzed in this study (N = 330 infant meconium and N = 217 mother fecal samples). For each infant, the hospital site of origin, sex and the gestational age category (28 to 30 weeks or >30 to 32 weeks) were recorded (Table S1). Sequencing data were generated for 665 samples (including 118 controls), with a mean of 23,792 reads per sample (median of 21,055 reads, 25^th^ percentile of 9,586 reads, 75^th^ percentile of 33,167 reads). The length distribution of ASVs was bi-modal, with peaks at 251 and at 200 nucleotides long. The longer length is the expected value for bacterial amplicons. The shorter ASVs were classified as human mitochondria using BLASTn and the NCBI nucleotide collection (nt).

10.1128/mSphere.00808-21.4 TABLE S1 Metadata for all samples used during this study. Download Table S1, CSV file, 0.08 MB. Copyright © 2022 Klopp et al. 2022 Klopp et al. https://creativecommons.org/licenses/by/4.0/ This content is distributed under the terms of the Creative Commons Attribution 4.0 International license.

The presence of bacterial genera known to be technical contaminants (*Delftia*, *Flavobacterium*, Pseudomonas, *Burkholderia*, *Sphingomonas*, *Corynebacterium*, and *Propionibacterium*) (23) was assessed and, except for *Corynebacterium*, all other known contaminants were found in less than 1% of samples and their 95^th^ percentile abundance was less than 0.1% of the meconium microbiomes. *Corynebacterium* was present in 26% of samples, with a median abundance of 0.2% in those samples.

**FIG 1:**
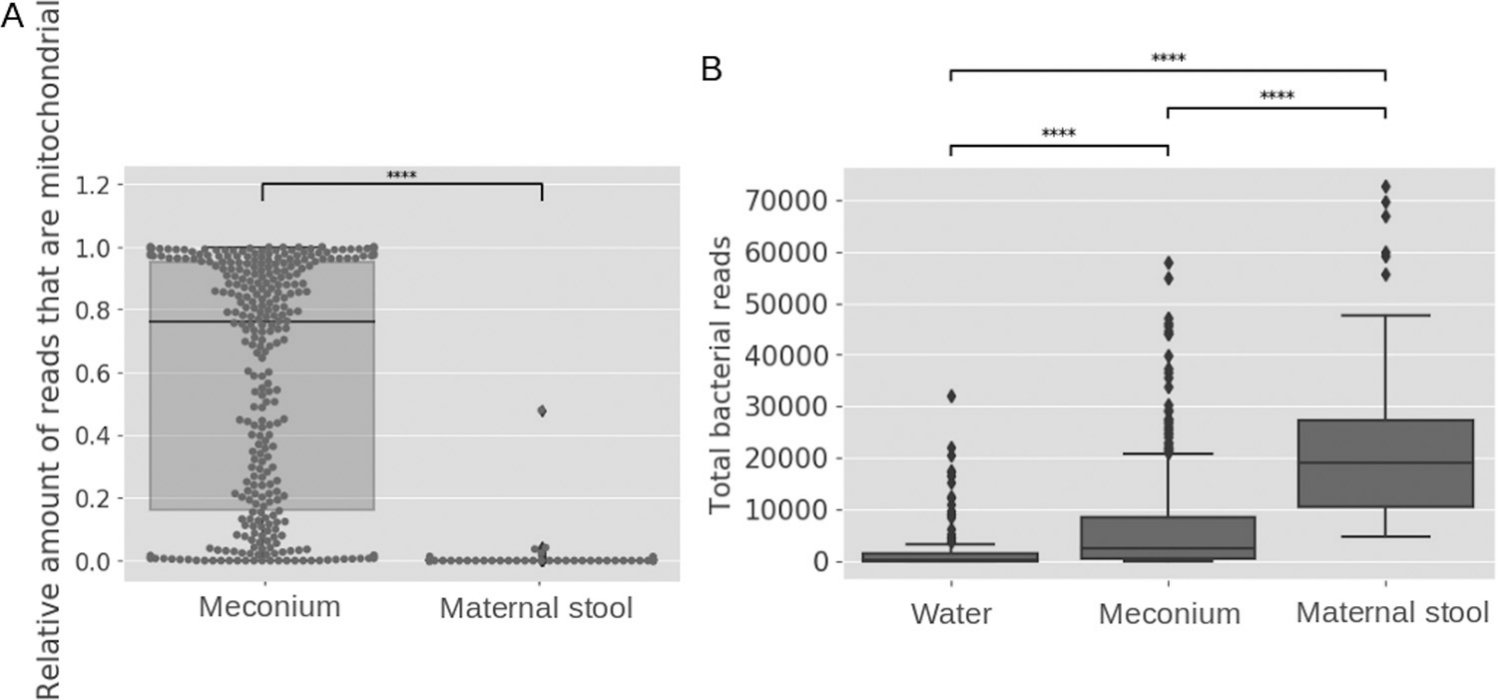
Differences in the yield of mitochondrial and bacterial sequences by sample type. (A) Proportion of total amplicon sequences that are mitochondrial sequences in infant meconium and maternal stool samples. *P* value annotation legend: ****: *P* ≤ 1.00e-04. (A) After removal of mitochondrial sequences, differences in the number of bacterial sequences in negative controls (“water,” N = 118), infant meconium samples (N = 330) and maternal stool samples (N = 217). Two mother samples and two meconium samples with more than 80,000 total bacterial reads were omitted from the plot for legibility (mothers: 93,954 and 131,844 reads, infants: 91,079 and 121,745 reads). Kruskal-Wallis tests were performed between all samples (A & B). *P* value annotation legend: ns: 5.00e-02 < *P* ≤ 1.00e + 00, *, 1.00e-02 < *P* ≤ 5.00e-02, **, 1.00e-03 < *P* ≤ 1.00e-02, ***, 1.00e-04 < *P* ≤ 1.00e-03, ****, *P* ≤ 1.00e-04.

The relative number of mitochondrial sequences was strikingly high in infant meconium samples relative to the maternal stool samples; with a median of 76% in meconium compared to 0% in maternal stool (Fig. 1A, Kruskal-Wallis test, *P* < 0.0001). To determine whether the high abundance of mitochondrial sequences was due to low bacterial load in samples, a defined bacterial load was added to a meconium sample prior to extraction. This led to a drastic reduction in the relative number of mitochondrial sequences in the microbiome and a microbiome composed of only spike-in DNA (Fig. S1). The amount of spike-in used would be expected to make up at most 10% of the microbiome from a normal stool sample.

10.1128/mSphere.00808-21.1 FIG S1 Rarefaction curve for meconium samples. Step width = 50. Download FIG S1, TIF file, 0.1 MB. Copyright © 2022 Klopp et al. 2022 Klopp et al. https://creativecommons.org/licenses/by/4.0/ This content is distributed under the terms of the Creative Commons Attribution 4.0 International license.

Before taxonomic analysis, quality control and removal of mitochondrial reads was performed. Based on negative controls and rarefaction curves (Fig. S1), samples were discarded if they contained less than 2500 bacterial reads after removal of mitochondrial sequences and filtration from the ASV identification. 165 meconium samples (50%) and 97 negative controls (82%) did not pass this threshold. Without mitochondrial sequences, the number of quality controlled bacterial reads in meconium samples was significantly higher than in the negative controls (Kruskal-Wallis test, *P* < 0.0001) and the number of bacterial reads in meconium samples was significantly lower than in maternal fecal samples (Kruskal-Wallis test, *P* < 0.0001) (Fig. 1B). Across all samples that passed all quality criteria (N = 165 infant meconium samples and N = 217 maternal samples), 6,267 ASVs were observed. Of these, 130 were identified as contamination with decontam, leaving 6137 ASVs. After decontamination, 469 ASVs had at least 1% abundance in at least two microbiome samples. In meconium samples, 2,312 ASVs were observed with 231 having at least 1% abundance in at least two samples. In mother samples, 4,538 ASVs were observed with 350 having at least 1% abundance in at least two samples.

### Taxonomic composition of infant meconium and maternal stool.

**FIG 2:**
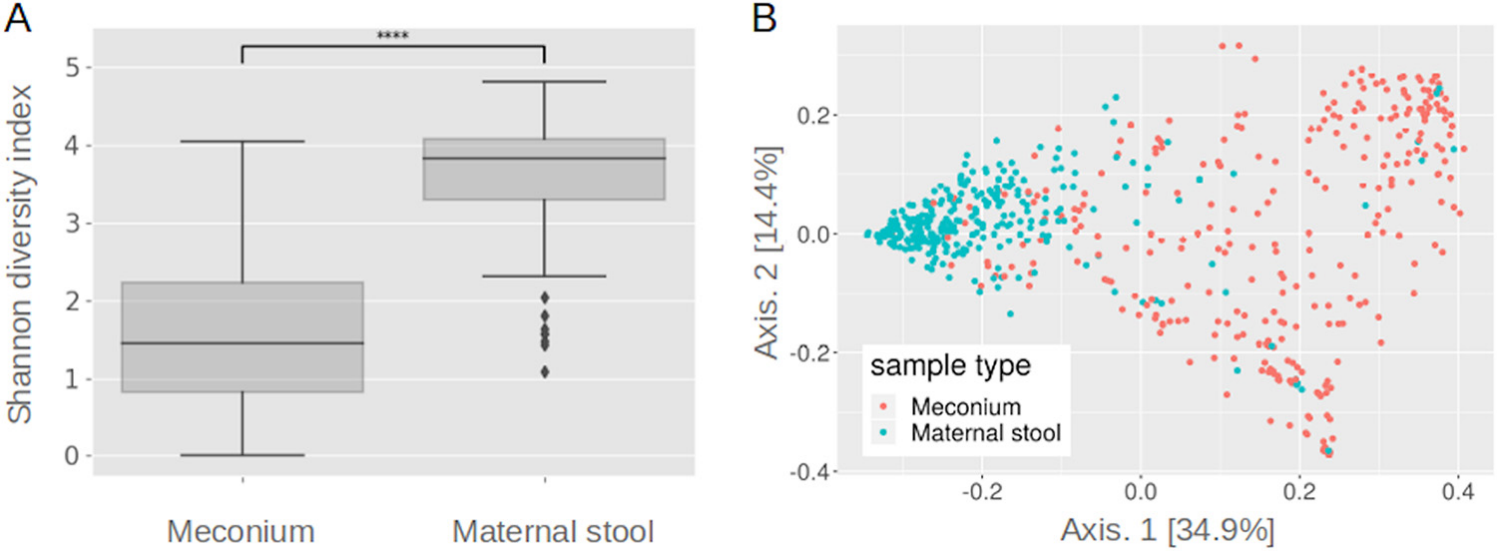
Diversity in meconium and maternal stool samples on the ASV level. (A) The difference in alpha diversity measured by the Shannon Index reveals that meconium samples have far less diversity than mother samples. *P* value annotation legend, ****, *P* ≤ 1.00e-04. (B) Beta diversity of infant meconium and maternal stool samples. Principal Coordinate Analysis (PCoA) based on the weighted UniFrac distances between samples is shown. Every point represents one sample and is colored based on the sample type.

16S rRNA gene-based microbiome taxonomic profiles were compiled from bacterial sequences for the samples that passed all quality criteria (N = 165 infant samples and N = 217 mother samples) (Tables S2, S3 and S4, https://seafile.rlp.net/d/37e022ec0ccb43018073/). As expected, significant differences in the microbiome composition of infant meconium and maternal stool samples were observed, (PERMANOVA *P* < 0.001). The alpha diversity was significantly higher in maternal stool samples than in meconium (Shannon index, Fig. 2A, Kruskal-Wallis test, *P* < 0.0001). If repeatedly subsampled to 2500 reads, meconium samples had a median of 12.5 ASVs per sample and maternal stool samples had a median of 112.5 ASVs per sample (meconium: 25^th^ percentile = 6 ASVs, 75^th^ percentile = 52 ASVs, ranging from 1 to 151 ASVs per sample, mean across subsampling; maternal stool: 25^th^ percentile = 77 ASVs, 75^th^ percentile = 145 ASVs, ranging from 5 to 253 ASVs). The diversity among mother samples was lower than the diversity among meconium samples, as seen in the spread of data in the PCoA (Fig. 2B, Permutation test for homogeneity of multivariate dispersions, *P* value < 0.001). There was no clustering of samples based on hospital sites (See Fig. S3).

10.1128/mSphere.00808-21.3 FIG S3 Beta diversity of infant and mother samples on the ASV level. Principal Coordinate analysis (PCoA) based on the weighted UniFrac distances between samples is shown. Every point represents one sample and is colored based on collection site. Download FIG S3, TIF file, 0.3 MB. Copyright © 2022 Klopp et al. 2022 Klopp et al. https://creativecommons.org/licenses/by/4.0/ This content is distributed under the terms of the Creative Commons Attribution 4.0 International license.

The taxonomic composition of meconium was significantly associated with the gestational age at birth and infant age when the meconium was collected (Table 1). Hospital collection site was also associated with composition of meconium and maternal stool samples, however, permutation tests for homogeneity of multivariate dispersions indicated that these differences might be due to differences in internal variation within the hospital sites rather than differences in taxa (Table 1).

**TABLE 1:** Association of gestational age at birth, age at meconium sample collection, and hospital collection site on overall microbiome composition (ASV level) in meconium from preterm infants

	Gestational age	Hospital site	Age at meconium collection
Permanova *P* value	*P* < 0.0001	*P* < 0.009	*P* = 0.028
Effect size	0.018	0.12	0.02
Dispersion test	*P* = 0.304	*P* = 0.001	*P* = 0.187

**FIG 3:**
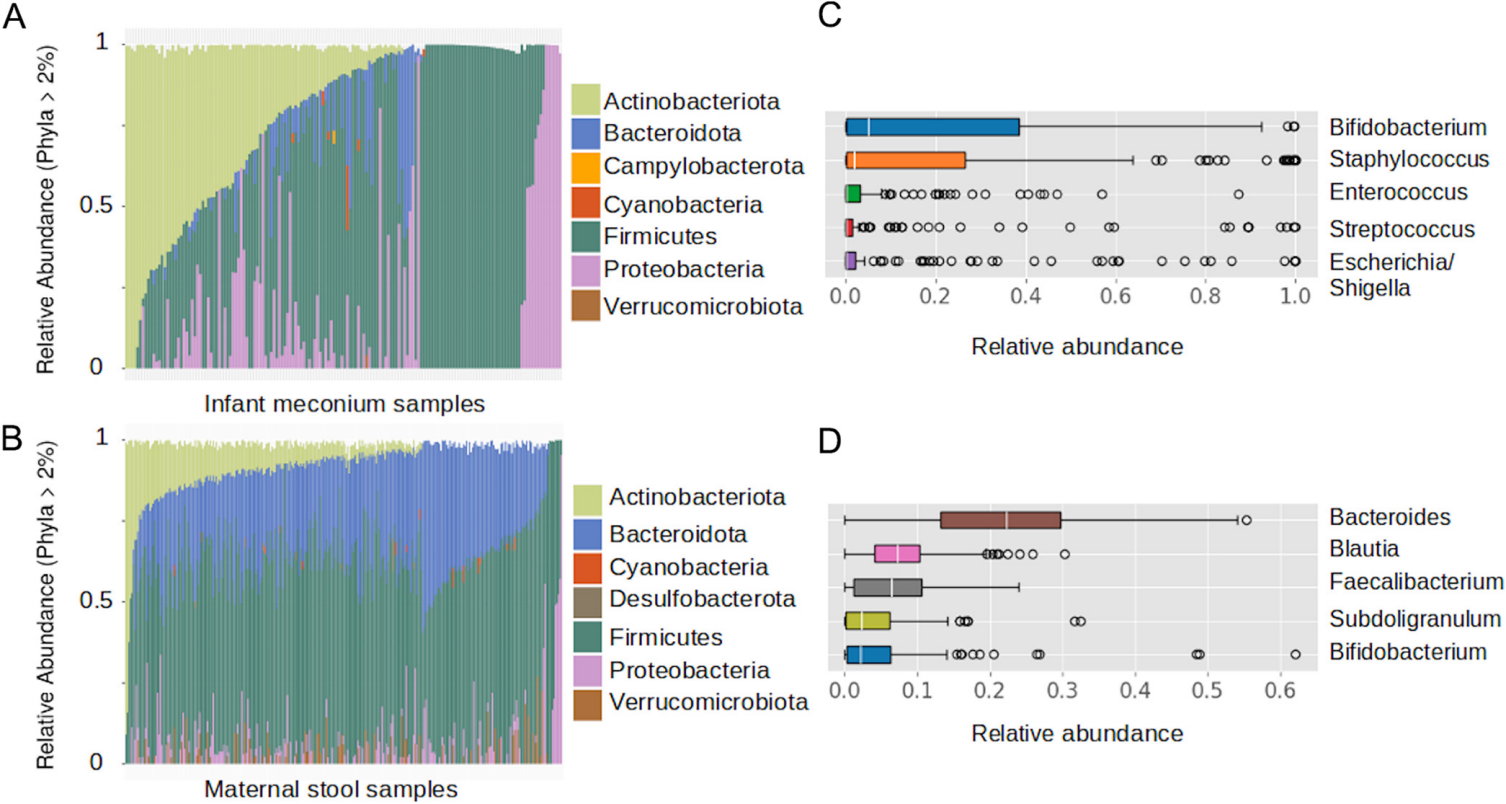
Taxonomic composition of the meconium and maternal stool samples. (A) Phylum composition of the infant meconium samples (N = 165). (B) Phylum composition of the maternal stool samples (N = 217). Only phyla that comprised more than 2% of total microbiome composition across samples are shown. (C) The five most abundant genera by median abundance across samples in meconium and (D) maternal stool microbiomes.

Maternal stool and meconium samples primarily consisted of bacteria that are members of the phyla *Firmicutes*, *Bacteriodota*, *Proteobacteria*, and *Actinobacteria* (Fig. 3A and B). At the genus level, the most common bacterial genera differed between mother and infant samples. The most abundant genus in meconium was *Bifidobacterium*, which was also commonly present in mothers. The other top genera in meconium (Staphylococcus, *Enterococcus*, Streptococcus, and Escherichia*-Shigella*) were also found in maternal stool samples but at much lower abundances (median: 0%, 0.04%, 0.72%, 0.12%, respectively). *Bacteroides* was the most common genus in maternal stool samples but did not constitute a major genus in the meconium samples (median: 0.04%) (Fig. 3C and D). A full list of genus and ASV abundances in meconium and maternal stool samples is depicted in Tables S6, S7, S8, and S9. Other top genera in maternal stool samples (*Blautia*, *Faecalibacterium*, and *Subdoligranulum*) were present in some of the meconium samples but at lower prevalence and abundance (prevalence: 38%, 37%, 28%, respectively, with median of 0% abudance and a 75^th^ percentile < 0.01% abundance in the meconium microbiome).

10.1128/mSphere.00808-21.5 TABLE S6 Statistics on the distribution and abundance of ASVs in infant meconium samples. Download Table S6, CSV file, 0.4 MB. Copyright © 2022 Klopp et al. 2022 Klopp et al. https://creativecommons.org/licenses/by/4.0/ This content is distributed under the terms of the Creative Commons Attribution 4.0 International license.

10.1128/mSphere.00808-21.6 TABLE S7 Statistics on the distribution and abundance of ASVs in maternal stool samples. Download Table S7, CSV file, 1.0 MB. Copyright © 2022 Klopp et al. 2022 Klopp et al. https://creativecommons.org/licenses/by/4.0/ This content is distributed under the terms of the Creative Commons Attribution 4.0 International license.

10.1128/mSphere.00808-21.7 TABLE S8 Statistics on the distribution and abundance of genera in infant meconium samples. Download Table S8, CSV file, 0.02 MB. Copyright © 2022 Klopp et al. 2022 Klopp et al. https://creativecommons.org/licenses/by/4.0/ This content is distributed under the terms of the Creative Commons Attribution 4.0 International license.

10.1128/mSphere.00808-21.8 TABLE S9 Statistics on the distribution and abundance of genera in maternal stool samples. Download Table S9, CSV file, 0.01 MB. Copyright © 2022 Klopp et al. 2022 Klopp et al. https://creativecommons.org/licenses/by/4.0/ This content is distributed under the terms of the Creative Commons Attribution 4.0 International license.

**FIG 4:**
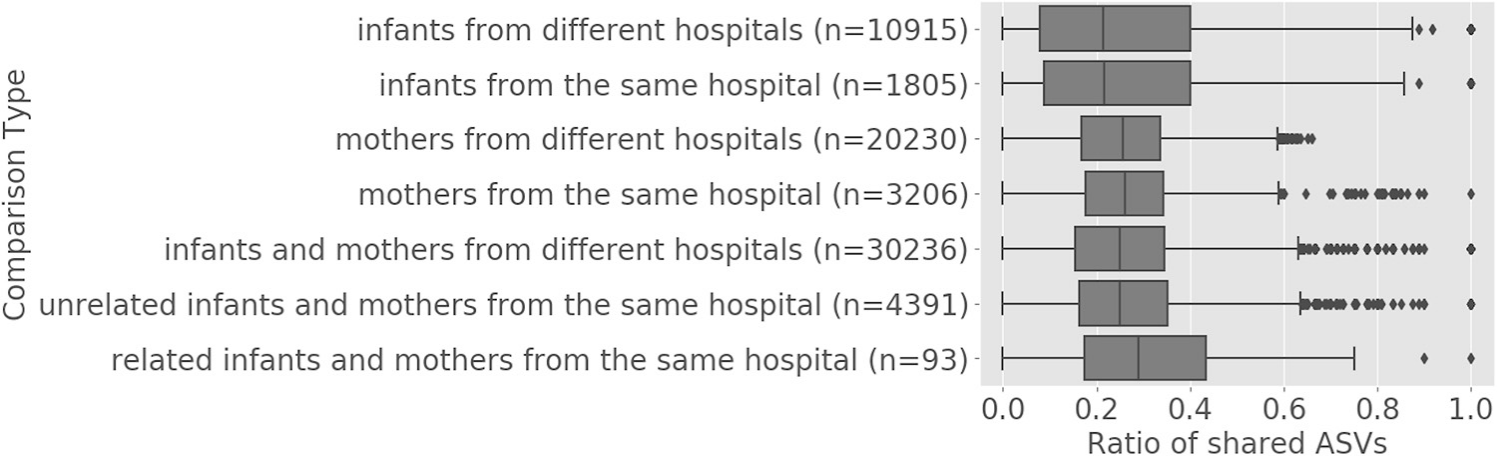
Proportion of shared ASVs in meconium and maternal stool samples. Proportion of shared ASVs was computed for different sample sets. Each boxplot shows one type of comparison. On the *y* axis the number (n) of pairwise comparisons for each comparison type is listed. No statistically significant differences between comparison types were found.

Meconium samples from infants and stool samples from their mothers were tested for shared taxa. Eighty-two mother-infant pairs were analyzed. A median of 28% of ASVs in the infant samples were also present in the corresponding mother samples, which was not significantly different from any other comparison between mothers and unrelated infants, either from the same or different hospitals (Kruskal-Wallis test, *P* > 0.05, with Bonferroni correction) (Fig. 4 and Table S5, https://seafile.rlp.net/d/37e022ec0ccb43018073/). Mother samples from the same hospital that shared 100% of ASVs were from the same mother that gave birth to twins, so was sampled twice. Only one meconium sample had complete presence of its ASVs in its corresponding mother sample and the infant in question only had 2 ASVs.

**FIG 5:**
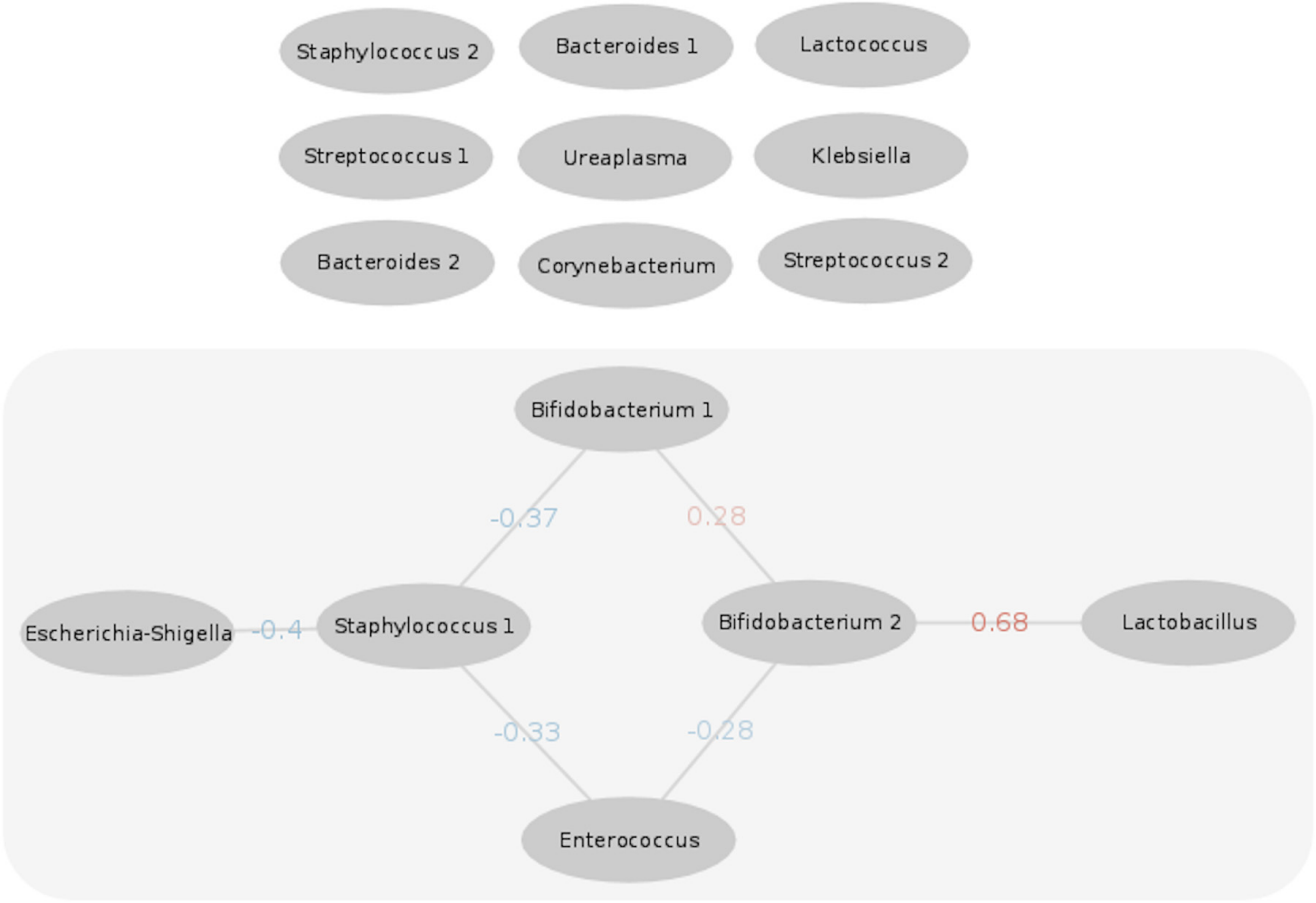
Co-occurrence network of the most abundant genera in infant meconium. Nodes are ASVs (taxa of approximately species-level resolution) with their respective genus classifications as labels. The 15 most abundant genera were selected according to median and mean abundance. Edges represent statistically significant correlations. The weight of the correlation is color coded from blue (negative correlation) to red (positive correlation). The significance level is alpha = 0.01.

Taxa across meconium samples were tested for co-occurrence. Out of the 15 most abundant genera (selected by median and mean abundance), significant correlations in abundances were observed for six taxa (discretized mutual information test as implemented by FlashWeave, alpha = 0.01, Fig. 5). Two ASVs (taxa of approximately species-level resolution) that were classified as members of the genus *Bifidobacterium* showed a positive co-occurrence and one of these is also highly correlated with the presence of *Lactobacillus*. Staphylococcus, and *Enterococcus* are each negatively correlated with a *Bifidobacterium* ASV, as well as to each other. The presence of a Staphylococcus ASV was negatively correlated with Escherichia*-Shigella*. Table 2 shows an overview of the prevalence and abundance of the correlated taxa. Nine of the 15 most abundant ASVs showed no significant correlation with each other. The ASV sequences of “*Bifidobacterium* 1” and “*Bifidobacterium* 2” (numbering is study specific) are 98% identical, with the latter likely belonging to Bifidobacterium animalis, based on nucleotide sequence similarity to reference sequences in NCBI nt database.

**TABLE 2:** Prevalence and abundance statistics and taxonomic classifications of bacterial ASVs in meconium samples that showed significant correlations in the co-occurrence network analysis

AASV	Prevalence	Median abundance	25^th^ percentile	75^th^ percentile
*Bifidobacterium* 1	38 %	3.6 %	0.04 %	31.91 %
*Bifidobacterium* 2	14 %	0 %	0%	0.12 %
*Lactobacillus*	17 %	0 %	0 %	0.16 %
Staphylococcus	75 %	2 %	0.05 %	12 %
Escherichia-*Shigella*	63 %	0.48 %	0 %	7 %
*Enterococcus*	63 %	0.8 %	0 %	5.4 %

### Scoring meconium suitability for bacterial sequencing.

The taxonomic analysis described above revealed that the primers used in this study amplified a product from the human mitochondrial 12S rRNA gene. The sequencing data showed that this product was consistently and distinctively shorter than the bacterial product (ca. 200 bp for mitochondrial amplicons versus ca. 250 bp for the bacterial amplicons). This difference can be leveraged to test for the presence of both products in samples prior to DNA sequencing. After extraction and amplification of DNA from meconium samples, five distinct patterns of amplicon length distributions per sample emerged when the DNA was subjected to gel electrophoresis. We classified these in a scoring system from one to five (Table 3), which we call a Meconium Bacterial Load (“MecBac”) score. Samples with a score of one include only the amplicon product that has the expected size from amplification from bacterial DNA. Samples with scores of two and three have some bacterial product, but also contain mitochondrial product. Scores of four and five do not support the presence of any bacterial product in the sample. 47% of the meconium samples in this study had a score of 3 or better, 53% were unsuitable for typical microbiome sequencing.

**TABLE 3:** Meconium bacterial load scoring system (“MecBac” Score) from 1 (best for microbiome sequencing) to 5 (worst for microbiome sequencing)

Meconium bacterial load score (“MecBac” score)	Gel band of size 200 bp (mitochondrial)	Gel band of size 250 bp (bacterial)	Suitability for bacterial microbiome sequencing	Percentage of meconium samples in this study
1	Absent	Present	Excellent	25 %
2	Present	Present	High	14 %
3	Present	Present (Lighter)	Reasonable	8 %
4	Present	Absent	Unsuitable	36%
5	Absent	Absent	Unsuitable	17 %

**FIG 6:**
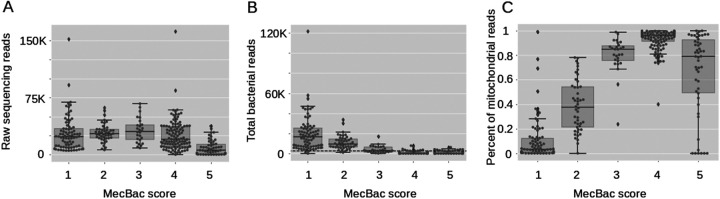
The quantities of raw, bacterial, and mitochondrial reads are associated with the meconium gel quality (MecBac) score. (A) Samples with the worst quality score tend to yield the fewest reads when sequenced. (B) Meconium with a better score yields higher numbers of high-quality bacterial reads. The dashed line shows the minimum threshold of 2500 reads. (C) A better (lower) MecBac score is correlated with a lower proportion of mitochondrial reads (Spearman correlation coefficient, 0.64, *P* < 0.0001, N = 327).

The quantity of bacterial reads per sample, after quality control and removal of mitochondrial sequences, correlates strongly with the MecBac score (“total reads” analysis [see methods], Pearson correlation coefficient: −0.61, *P* < 10^−34^, Spearman correlation coefficient: −0.78, *P* < 10^−67^, N = 327). Samples that were classified with the best score (1) had the most bacterial reads but not the most total overall reads (Fig. 6).

MecBac scores were not correlated with the gestational age of infants at birth or the hospital site (Spearman correlation coefficient: −0.08, *P* > 0.15, 0.02, *P* > 0.72, N = 327) but showed an inverse correlation with the age of the infants when the meconium was collected (Spearman correlation coefficient −0.358, *P* < 0.001, N = 241). Samples collected later in life (after more time has passed for bacterial growth) had a better MecBac score. After removal of contaminants and mitochondrial sequences, the composition of meconium bacterial communities was significantly associated with a sample’s MecBac score, after controlling for gestational age and hospital (PERMANOVA, *P* < 0.001, effect size = 5.8%). There were also significant differences in alpha diversity between samples with different MecBac scores (Kruskal-Wallis test, *P* < 0.00001). The samples with the best score tended to have lower alpha diversity. The higher diversity in samples with worse MecBac scores could be due to their lower bacterial loads, which increases the likelihood of contamination and random amplification of this DNA or it could be a real biological difference, whereby samples that have a lower bacterial load also tend to have a different community composition. Samples with the best MecBac score (1) had significantly fewer sequences identified as contamination during the decontamination step than other meconium samples (Kruskal-Wallis test, *P* < 0.00001).

## DISCUSSION

The meconium microbiome has been discussed as a potential source of information about long term health and disease (13). Recent evidence demonstrates that the meconium microbiome may predict and impact health and disease both early in life, in terms of weight gain and growth (17), sepsis risk (24), and NEC propensity (25), as well as later in life, e.g., concerning the incidence of asthma, allergies, obesity and psychiatric illnesses (26). A comprehensive understanding of baseline bacterial structure and composition of meconium may complement our knowledge in this respect. In this study, we provide a basic characterization of the meconium microbiome in 330 infants and comparisons with a subset of their mothers. These findings are representative of very preterm infants in Germany. Preterm infants may have a different meconium composition than full term infants as passage takes longer and is often delayed in preterm infants (27). As preterm infants are at increased risk of poor health outcomes, possibly linked to gut dysbiosis (28), gut microbiome studies are valuable in this population to understand mechanisms and find new targets for preventive and therapeutic strategies.

Since meconium samples contain very small amounts of bacterial biomass, it is especially important to assess possible contamination (19, 20) and to avoid spurious results by using negative controls and purpose-built statistical methods (29). To only consider high quality microbiome profiles in our analysis, we included negative and positive controls and used specific software to identify likely contamination. Samples that contained fewer bacterial reads than the mean in the negative controls were discarded. Any bacterial community in these samples was not considered to be well represented by the typical amplicon sequencing approach used here. Interestingly, about half of all meconium samples in this study did not pass this threshold and had a high abundance of mitochondrial sequences. This finding suggests that the bacterial load in meconium varies greatly across infants, with many having a smaller amount than is accessible to study using typical amplicon microbiome approaches. This variation appears to be correlated with the age of the infant, such that meconium passed later in life (e.g., at day 2) tends to have more bacterial load than meconium passed earlier in life (e.g., at day 1). This trend fits with the hypothesis that meconium is sterile at birth and then is colonized postnatally. This correlation is weak however, so there are likely other factors that also impact the bacterial load, such as prolonged rupture of membranes, mode of birth, diet and environmental exposure. This variation in bacterial presence has been previously reported in another much smaller study (N = 15) (30), and hints at a possible reason for the ongoing controversy (31) on the presence and source of bacteria in meconium.

In our study, the main driver of DNA load in many meconium samples was human mitochondrial DNA (comprising a median of 76% of amplicon reads) instead of bacterial DNA. As the first pass stool, such abundance of mitochondrial DNA is not unexpected as meconium contains many human cells such as intestinal epithelial cells of the infant. However, a PCR using 16S rRNA gene specific primers should not result in the amplification of these mitochondrial sequences. We speculate that the widely used protocol of 16S primer pairs 515f and 806r, first published by Caporaso et al. (32) and later optimized (33) might have led to this spurious amplification. The forward primer has a sequence identity of 85.71% and the reverse primer of 89.47% to the 12s rRNA gene of the human mitochondrium spanning a 199 bp long segment, explaining the gel band at around 200 bp in our study. In fecal samples with a sufficient amount of bacterial DNA the competition is large enough to inhibit the primer binding to mitochondrial DNA (Fig. 1B). However, in a very low biomass setting like meconium, imperfect binding to the mitochondrial DNA might facilitate their amplification. This hypothesis is further strengthened by the observation that the relative amount of amplified mitochondrial DNA is drastically reduced when using spike-in controls of defined bacterial load (Fig. S2), suggesting very low or absent bacterial biomass in the original sample.

10.1128/mSphere.00808-21.2 FIG S2 Meconium microbiome of one exemplary sample that has been sequenced in its unaltered state as well as in duplicate with 2 μl and 10 μl of ZymoBIOMICS Spike-in Control I (consisting of Imtechella halotolerans, Family Flavobacteriaceae and Allobacillus halotolerans, Family Bacillaceae) added, respectively. Download FIG S2, TIF file, 0.03 MB. Copyright © 2022 Klopp et al. 2022 Klopp et al. https://creativecommons.org/licenses/by/4.0/ This content is distributed under the terms of the Creative Commons Attribution 4.0 International license.

Interestingly, other studies investigating the meconium microbiome composition using the V4 region of the 16S rRNA gene such as one from China (18) and one from Brazil (17) found *Proteobacteria* to be a highly abundant member of the microbiome in most samples, while we found it only sporadically (Fig. 3A). Using the taxonomic classification provided by SILVA, human mitochondrial amplicon sequences are classified as Kingdom: *Bacteria*, Phylum: *Proteobacteria*, Class: *Alphaproteobacteria*, Order: *Rickettsiales*. It might thus be possible that mitochondrial sequences introduce bias to the structure of underlying microbial communities. However, it is well known that other covariates like geographical origin of the samples (34) and differences in extraction methods (especially when working with low biomass samples such as meconium [35]) might explain microbial differences found in the infant gut.

In the literature, the early ecological succession in the infant gut is described as being driven by niche-based competition after an initial seeding with bacteria whose taxonomic composition is in part subject to stochastics effects (36). Evidence for these stochastic elements can be seen in the large beta diversity between meconium samples in this study. Similarly to full term infants (37), the genus *Bifidobacterium* was one of the main genera in the meconium of premature infants. This highlights the pivotal role of this genus in the developing infant gut microbiome from birth. Microbiomes of preterm infants also tend to show higher relative abundances of Staphylococcus immediately after birth compared to full term infants, which is consistent with our data (38). In line with earlier studies (39, 40), the meconium composition in our study is characterized by lower alpha diversity compared to the later microbiomes in preterm infants.

Mitochondrial amplification from the V4 primers led to most meconium samples having sufficient DNA concentration to pass typical sequencing library preparation quality controls (based on Qubit measurements). However, after removing mitochondrial sequences, 50% of samples lacked sufficient bacterial data for analysis and therefore wasted significant resources. Typical quality control procedures such as NanoDrop or Qubit measurements would not differentiate between bacterial and mitochondrial amplicon products and thus not predict these wasted samples. It is possible that this issue would not be as severe if using other 16S amplicon primers that targeted another region on the 16S rRNA gene. However, the V4 region of the 16S rRNA gene used in this study has substantial advantages over using other or multiple 16S rRNA regions in microbiome studies, such as a very low error rate (41) and suitability for meta-analysis, due to its widespread popularity in the field. To support future use of V4 primers to study meconium despite the nonspecific amplification issue, we developed a five-point scoring system to assess the suitability of meconium samples for bacterial sequencing (“MecBac” score) based on a simple gel electrophoresis of amplicon products. This allows the selection of promising samples prior to sequencing, which, based on the score distribution in our study, would potentially save 50% of the sequencing costs in future studies. While developed for 16S rRNA sequencing, this scoring system can also be used for sample selection for more expensive sequencing, such as metagenomic whole-genome shotgun sequencing.

For the infants in our study that had a high number of bacterial reads, the alpha diversity was sometimes especially low (Shannon index close to 0), indicating the dominance of only one bacterial genus or species. 35 infants were dominated by less than 5 ASVs. The main genera present in these microbiome samples were Staphylococcus, Escherichia*-Shigella*, and Streptococcus. All of these genera have members that are well known human pathogens, such as methicillin-resistant Staphylococcus aureus (MSRA), certain serotypes of Escherichia coli and many *Shigella* species that are the cause of shigellosis. The microbiome of the infants concerned might thus be in a state of extreme dysbiosis. However, two limitations of the 16S rRNA gene sequencing approach make it hard to draw definite conclusions. First, the lack of sufficient taxonomic resolution to identify bacteria below the genus level does not allow the discrimination of pathogenic and nonpathogenic strains in the aforementioned genera. Second, it is possible that the apparent abundance of bacterial species was artificially inflated through PCR kinetics early in the amplicon methodology. To validate these findings in future studies, other methods, such as targeted qPCR or amplification-free metagenomics, would be required.

The statistically significant co-occurrence correlations among abundant taxa in the meconium microbiome may provide insight into microbial interactions. Two ASVs belonging to the *Bifidobacterium* genus, which is well known to be a beneficial member of the early microbiome in infants (42), were negatively correlated with the potentially pathogenic genera Staphylococcus and *Enterococcus.* These negative correlations might be further evidence for the competition-based mechanism hypothesis of *Bifidobacterium* in the clinical effectiveness of probiotics (43). Several molecular mechanisms are known by which *Bifidobacterium* and other probiotic bacteria can have suppressing effects on intestinal pathogenic bacteria (reviewed in van Zyl et al. [44]). Certain species of the genus *Bifidobacterium*, for example, are able to produce proteins that actively prevent pathogenic bacteria from attaching to the mucosal cells of the host, produce unique bacteriocins, called bifidocins, or simply competitively outperform pathogens in the struggle for resources (44). The strong positive correlation between *Bifidobacterium* and *Lactobacillus* is in line with network analyses of the gut microbiome from other groups (45) and these two genera are also known to be abundant in the gut microbiomes of term infants (46) especially after vaginal delivery (47). In our study, these taxa were included in a probiotic given to half of the infants. Since the study is still blinded, we could not assess in detail to what extent the probiotic could be strengthening or even driving the association. Unfortunately, the resolution of 16S rRNA gene sequencing does not always allow the differentiation of different species within a genus. Thus, the identity of the *Bifidobacterium* and *Lactobacillus* cannot be unambiguously assessed for all ASVs at a level lower than genus. However, since the genus *Bifidobacterium* is present in 76.8% of samples while only 50% of the infants received a probiotic, it is reasonable to assume that naturally occurring *Bifidobacterium* are highly relevant to the detected associations in the meconium samples. In future studies aimed at understanding dysbiosis in infants, the interactions between different taxa in the normal microbiome between presumably beneficial bacteria (*Bifidobacterium*, *Lactobacillus*) and potentially pathogenic bacteria should thus be given a pivotal role.

Earlier studies on the transfer of bacteria from mothers to full term, nonhospitalised infants found that the gut microbiome is the major source of shared strains (48) and that up to 63% of the infant’s microbiome can be explained by bacteria in the maternal gut (49). Mother-infant pairs were characterized by an increased number of shared ASVs, compared to unrelated pairs. However, probably due the sample size of this study, this difference was not significant. This indicates that while a transfer might have occurred, we do not have evidence that it was the main source for bacteria in the meconium. However, insufficient taxonomic resolution prohibits final conclusions on the exact transfer of bacteria from mothers to infants. Future studies would require strain level information to make these conclusions, such as from metagenomic whole-genome shotgun sequencing.

### Summary and conclusion.

Analysis of meconium, the first pass stool of newborns, offers insights into the very early development of the human gut microbiome. Characterization of the microbiome composition of very preterm infants from 15 geographically dispersed German hospitals revealed a large variability in the level of detectable bacteria across meconium samples, ranging from high abundance of bacteria to undetectable bacterial loads. Bacterial load was weakly correlated with the time span between birth and sample collection. This is in line with the hypothesis that meconium is sterile in the womb and is colonized postnatally. In low bacterial mass samples, standard 16S rRNA methodology amplified mitochondrial DNA instead, which can be misclassified as Proteobacteria using standard analytical approaches. Through exclusion of these mitochondrial sequences and *in silico* removal of likely contaminants, identified by using negative controls, we produced robust bacterial profiles of meconium. These profiles revealed that meconium shows lower alpha diversity than adult stool and is often dominated by just a few species. Common taxa in meconium include known infant associated but also pathogen-containing genera (*Bifidobacterium*, Staphylococcus, *Enterococcus* and Streptococcus, respectively). *Bifidobacterium* was negatively correlated with pathogen-containing genera, providing further support for their role as beneficial bacteria. Due to the large range of bacterial load in meconium samples, from essentially absent to abundant, bacterial sequencing effort is easily wasted on many samples. To make progress in this respect, we developed a five-level scoring system based on simple gel electrophoresis (“MecBac” score) that allows identification of promising samples prior to sequencing. This system, as well as the foundational meconium characterization provided in this study, should be helpful for future studies of the meconium microbiome, in particular for higher resolution techniques, such as metagenomic sequencing.

## MATERIALS AND METHODS

### Sample collection.

The samples collected in this study are part of the PRIMAL study. The study protocol describing in detail the infant population, sample collection and storage, exclusion and inclusion criteria and implementation has been published (21). In short, very preterm infants (VPI, gestational age 28–32 weeks) were recruited from 15 German pediatric hospitals. After written consent was obtained from the parents, meconium samples from the infants and fecal samples from the mothers were collected, transported on dry ice in an anaerobic environment, and stored at −80 Celsius until further processing. Maternal stool samples were collected in 4 hospitals (Freiburg, Heidelberg, Homburg, and Lübeck) within the first 7 days after delivery. Meconium samples were collected with a median time of 2 days after birth (25th percentile: 1 day, 75th percentile: 3 days). 57.5% of the infants were male and 42.5% were female. Exactly 50% of infants were born in the gestational weeks 28 to 30 and in >30 to 32, respectively. Meconium samples were analyzed for color and texture and only samples that had typical meconium characteristics (dark color, sticky and tar-like) were considered for this study. Half of the infants received a probiotic supplement (Probactiol infantis) prior to meconium collection, consisting of Bifidobacterium animalis *subsp. lactis*, (BB[1]12), *B. infantis* and Lactobacillus acidophilus (La-5) each at 1.5*10^9^colony forming units. The present study represents a pilot analysis of the wider PRIMAL cohort using only the early life (meconium) time points and is still blinded in regard to probiotic supplementation. By the preregistered protocol of the PRIMAL RCT, unblinding the data are scheduled to take place only once endpoints are reached, ensuring methodological rigor and adherence to regulatory requirements. Institutional review board approvals have been obtained at all participating sites.

### DNA extraction and 16S rRNA gene sequencing.

Positive (N = 5) and negative (N = 118) control samples were used to monitor sample contamination. ZymoBIOMICS Microbial Community DNA Standard was used as a positive control. Every step described below was accompanied by sterile, nuclease-free, DEPC-treated water as negative controls.

DNA was extracted from meconium and maternal stool samples using the DNeasy PowerSoil Pro kit (Qiagen, Germany) including a bead-beating step at 30 Hertz for 2*7 min on a TissueLyser (Qiagen, Germany). The V4 region of the 16S rRNA bacterial genes was targeted for amplification with primers (forward: 5′-GTGCCAGCMGCCGCGGTAA-3′ 515F, reverse: 5′-GACTACHVGGGTWTCTAATCC-3′ 806R) (32). Gel electrophoresis was used to assess the approximate quantity and length of the amplicons. Gel bands of the shorter than expected amplification products (200 bp) were cut out, sequenced and classified as mitochondrial sequences using the BLASTn algorithm (50) with the NCBI nucleotide collection (nt) database.

All products were sequenced on a MiSeq (Illumina, Inc., USA) using V2 chemistry producing 2 × 250 bp paired-end reads. Samples were sequenced over 5 sequencing runs, each with positive and negative controls. To approximate the absolute cell count in one sample, 2 μl and 10 μl ZymoBIOMICS Spike-in Control I (High Microbial Load) were added to two aliquots of the same sample, respectively, and sequenced as separate samples. The spike-in control is composed of a known quantity of DNA from Imtechella halotolerans and Allobacillus halotolerans. For normal fecal samples, Zymo Research suggests using 20 μl of ZymoBIOMICS^TM^ Spike-in Control I to allow the quantification of bacterial cells in the sample. This added DNA, usually accounts for less than 10% of the resulting microbiome after analysis (51). Bacterial load estimates were based on PCR band results.

### Bioinformatic analysis.

Resulting sequencing data were processed using the R statistical environment (version 3.4.4) (52). Two different analyses were performed using the pipeline described below. One analysis in which all sequences were kept, including mitochondrial reads (“total reads”), and one in which all sequences much shorter than the expected amplicon length of 249–254 bp (i.e., mitochondrial sequences) were discarded (“bacterial reads”). Samples with 0 reads were not considered for the following analysis.

The DADA2 package (53) was used to quality filter the reads, trim off the primer sequences and truncate reads to 240 bp. This truncation step was skipped in the “total reads” analysis. Unpaired reads and reads that did not pass quality control were discarded. One error model per sequencing run was trained to infer exact amplicon sequence variants (ASVs). Forward and reverse reads were merged and chimeric sequences removed. ASVs were taxonomically classified using the RDP Naive Bayesian Classifier (54) and the SILVA high quality rRNA database version 138 (55). The ASVs were then decontaminated using the decontam package version 1.1.2 (29). This algorithm statistically assesses if an ASV is likely to be technical contamination considering its presence in negative-control samples and the DNA concentration in the samples, assuming that contaminating sequences have a higher frequency in low-concentration samples. Only ASVs that had a less than 5% chance of being contaminants were kept for further analysis.

Subsequent data handling, processing and visualization was performed in the python programming language (version 3.7.3) (56) with a suite of data science packages (pandas (57), SciPy (58), numpy (59), seaborn (60), matplotlib (61), networkx (62)) provided in the anaconda (version 4.9.2) environment (63). Statistical analysis, including alpha and beta diversity calculations (Shannon diversity index and weighted Unifrac distances) and Principal Coordinate Analysis (PCoA) were performed in R using the phyloseq package version 1.22.3 (64). Permutational multivariate analysis of variance (PERMANOVA) was performed with the vegan package (version 2.5.6) (65) in R using the adonis2 function. Collection dates of meconium that were unusually high (>7 days) or not recorded were not used for the multivariate analysis of the influence of age at collection on the microbiome. Differential abundance analysis between groups was performed with the Mann-Whitney U test implemented in SciPy and multiple testing correction was performed using the Benjamini-Hochberg procedure. The co-occurrence network analysis was performed with FlashWeave (66) in the Julia programming language (version 1.6.1).

### Data availability.

The data sets generated and analyzed during the current study are available in the ENA repository under accession no. PRJEB47767. Metadata, including demographic and clinical data, are available in this article’s supplemental material files.
