# Supplementary figures and images for: Meconium Microbiome of Very Preterm Infants across Germany
Source: mSphere. 2022 Jan 12;7(1):e00808-21. doi: 10.1128/msphere.00808-21 (PMC8754166; doi:10.1128/msphere.00808-21)

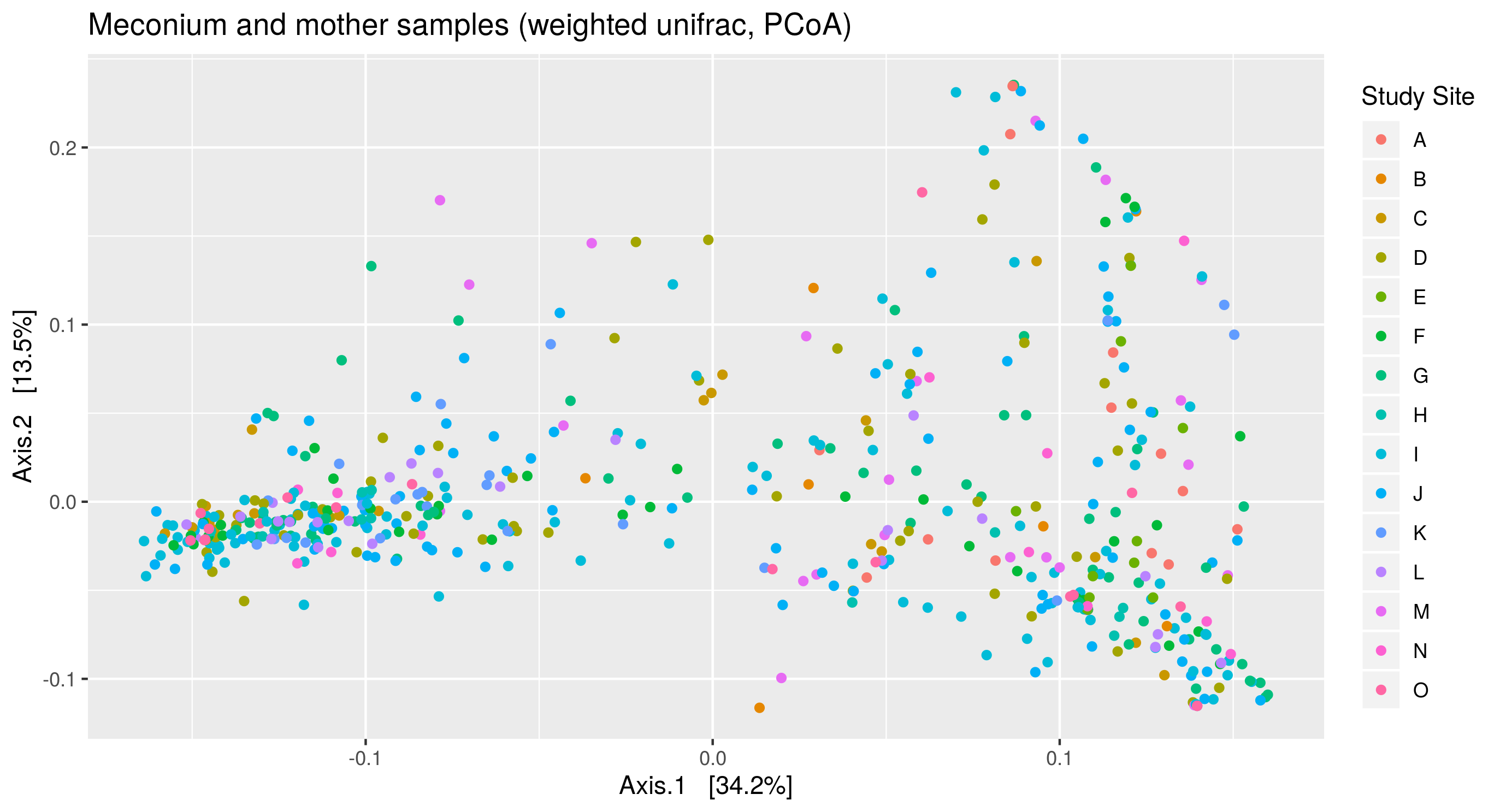

Supplement: FIG S3 [file msphere.00808-21-s0006.tif]

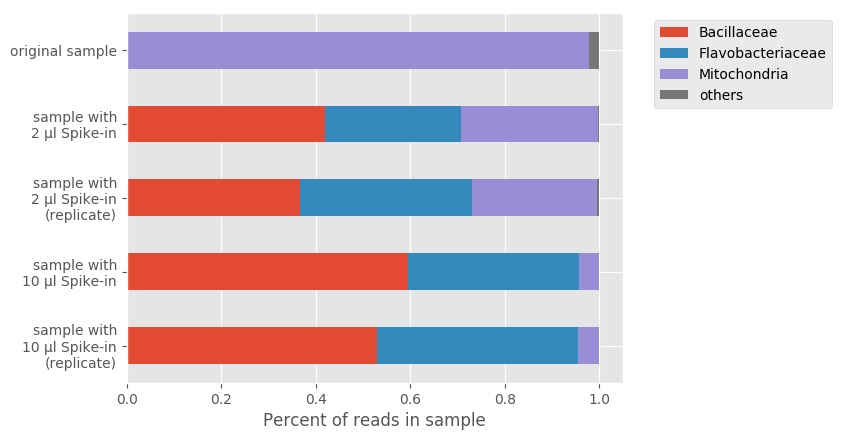

Supplement: FIG S2 [file msphere.00808-21-s0005.tif]
